# Laparoscopic Radical Hysterectomy Results in Higher Recurrence Rate Versus Open Abdominal Surgery for Stage IB1 Cervical Cancer Patients With Tumor Size Less Than 2 Centimeter: A Retrospective Propensity Score-Matched Study

**DOI:** 10.3389/fonc.2021.683231

**Published:** 2021-06-10

**Authors:** Xiaoyue Chen, Jiangtao Yu, Hongqin Zhao, Yan Hu, Haiyan Zhu

**Affiliations:** ^1^ Department of Gynecology, Shanghai First Maternity and Infant Hospital, Tongji University School of Medicine, Shanghai, China; ^2^ Department of Gynecology, The First Affiliated Hospital of Wenzhou Medical University, Wenzhou, China

**Keywords:** laparotomy, prognosis, radical hysterectomy, survival, cervical cancer, laparoscopy

## Abstract

**Objective:**

To compare the oncologic outcomes between laparoscopic and open radical hysterectomy in patients with stage IB1 cervical cancer lesion less than 2 cm.

**Methods:**

Patients diagnosed FIGO (2009) stage IB1 (tumor diameter <2 cm) and underwent radical hysterectomy in our hospital between March 2008 and November 2018 were studied. A propensity-matched comparison (1:2) was conducted to minimize selection biases. Demographic and baseline oncologic characteristics were balanced between groups. Overall survival (OS) and disease-free survival (DFS) were assessed using the Kaplan–Meier model, along with univariable and multivariable regression analysis.

**Results:**

A total of 261 patients were enrolled in this study after propensity-matching, with 174 in the open group and 87 in the laparoscopic group. Disease relapsed in seven patients in laparoscopy group, and the recurrence rate was 8.0% (7/87). There were eight patients underwent abdominal radical hysterectomy experienced recurrence, and the recurrence rate was 4.6% (8/174). The multivariate analysis model revealed that laparoscopic operation was associated with higher risk of recurrence than abdominal radical hysterectomy (HR, 3.789; 95% CI, 1.143–12.559; *p* = 0.029). There were five patients or 2.9% (5/174) died in open surgery group and the corresponding percentage in laparoscopy group was 2.3% (2/87). No difference was found in OS between the two groups (HR, 1.823; 95% CI, 0.2673–12.44; log-rank *p* = 0.5398). All the recurrence occurred within two years after operation in the laparoscopy group, among which pelvic recurrence (85.7%) was dominant.

**Conclusion:**

Traditional laparotomy radical hysterectomy has a lower recurrence rate when compared with laparoscopic operation in those cervical cancer patients with a foci diameter less than 2 cm. However, no detrimental effect on survival was found in minimal invasive operation group. Further multi-center prospective trials are needed to confirm our results on a large scale.

## Introduction

Cervical cancer is a disease that is curable with early diagnosis and intervention, yet it remains the fourth leading cause of cancer-related death worldwide (1). Radical hysterectomy (RH) with bilateral pelvic lymph node dissection represents the first-line treatment for early-stage cervical cancer (2). The advantages and disadvantages of laparoscopic RH are controversial since the first case of a laparoscopic RH and paraaortic and pelvic lymphadenectomy was performed to treat a stage IA2 carcinoma of the cervix (3). Laparoscopic RH has gradually emerged as an alternative procedure for cervical cancer treatment in the last decade in China due to the improved laparoscopic equipment and accumulated experience and expertise of oncologists. More importantly, previous studies showed that patients could benefit from laparoscopic surgery with similar survival outcomes (4–8) as those, who underwent laparotomy, but had better life quality after the operation (9, 10).

The published result of Laparoscopic Approach to Carcinoma of Cervix (LACC) trial challenged the oncologic safety of minimally invasive radical hysterectomy and endorsed open surgery. The phase 3 trial indicated that minimally invasive radical hysterectomy had lower disease-free survival as well as higher local recurrence rate than open surgery (11). Meanwhile the postoperative life quality was similar between the two groups (12). The NCCN Clinical Practice Guidelines thus regarded open surgery as the standard approach for radical hysterectomy since 2019 (13).

However, LACC trial has its limitations. It cannot be generalized to patients with tumor size <2 cm, as it was not powered to evaluate the oncologic outcomes of the two surgical approaches in this context (11). So far, few articles directly explored the benefits of laparoscopic RH in cervical cancer with a foci diameter less than 2 cm (14–17).

The primary purpose of this propensity-matched retrospective observational analysis is to evaluate the oncologic outcome between laparoscopic RH and open surgery in cervical cancer patients with a lesion <2 cm. The highlight of this study is that all lesions were assessed by preoperative imaging exam, which were more practical in clinical practice. The findings of this study contribute to the growing body of evidence against the use of minimally invasive surgery for cervical cancer.

## Materials and Methods

### Sample Collection

This is a retrospective observational study. Cervical cancer patients, who were diagnosed and treated at the Division of Gynecology of The First Affiliated Hospital of Wenzhou Medical University between March 2008 and November 2018, were considered for our study. The criteria of choosing patients to be included in this study were as follows (1): histological diagnosis of adenocarcinoma, squamous cell carcinoma, or adenosquamous carcinoma of the cervix (2), age between 18 and 70 years old (3), International Federation of Gynecology and Obstetrics (FIGO) 2009 clinical stage IB1 with tumor size <2 cm and limited to the cervix (4), normal renal, hepatic, and cardiac function, and (5) signed informed consent and compliance to follow-up. The exclusion criteria were as follows (1): patients underwent vaginal radical hysterectomy or fertility-sparing procedures, and (2) synchronous malignancies in 5 years. The study was approved by the First Affiliated Hospital of Wenzhou Medical University Institutional Ethics Committee for Non-Interventional Research.

### Surgical and Perioperative Management

Primary preoperative evaluation consisted of a complete medical history, physical examination, laboratory examinations, electrocardiogram, pelvic ultrasonography, chest X-ray, pelvic computed tomography (CT)/magnetic resonance imaging (MRI) or positron emission tomography- computed tomography (PET-CT). Preoperative imaging assessment confirmed that there were no extrauterine or lymph node metastasis. Prior to surgery, all patients underwent mechanical bowel preparation and antibiotic prophylaxis. Deep venous thrombosis prophylaxis with low-molecular-weight heparin were performed according to Caprini Risk Assessment Scale for high risk of thromboembolism (12 h before surgery and postoperatively for 4 weeks). According to the NCCN guideline, all patients underwent type C radical hysterectomy with bilateral pelvic lymphadenectomy (18, 19). All procedures were performed by skilled surgeons. The uterine manipulator used in laparoscopic RH was a modified metal uterine manipulator. There were no significant differences in the facilities available for patient care. Adjuvant treatment was recommended, according to the National Comprehensive Cancer Network (NCCN) guidelines. Adjuvant radiation therapy was suggested according to Sedlis criteria, while chemo and radiation therapy was suggested in case of positive nodes, parametrial involvement, or positive surgical margins.

### Data Collection

All medical records were reviewed simultaneously by three trained residents, and independently checked by two experts to ensure the accuracy.

Patients were followed up 1 month and then every 3 months during the first 2 years after surgery, and twice a year afterwards. At each scheduled follow-up visit, pelvic examination and squamous cell carcinoma antigen (SCC) (for squamous and adenosquamous cancer) or carbohydrate antigen 125 (CA125) (for adenocarcinoma and adenosquamous cancer) were performed. Pelvic and chest CT were tested once a year. The median follow-up time was calculated from the date of surgery. A secretary made periodic phone call to patients before scheduled outpatient follow-up visit to reduce omitted follow-ups. Dates and sites of recurrence were recorded.

Staging system and architectural grade were reported according to the FIGO statements. The World Health Organization (WHO) taxonomy was used to classify histologic subtypes. Tumor size was defined as the largest diameter of the lesion in preoperative imaging evaluation according to pelvic MRI or CT. DFS was defined as the interval from the operation to the first finding of any recurrence or last follow-up visit. OS was defined as the interval from the operation to the cervical cancer related death or last follow-up visit.

### Statistical Analysis

Patients underwent laparoscopic radical hysterectomy (LRH) were matched 1:2 to those underwent open abdominal radical hysterectomy (ARH). Six baseline characteristics (age, histology, parametrial involvement, lymphovascular space invasion, pelvic lymph nodes, surgical margin, and depth of cervical stromal invasion) were selected as covariates in propensity score match model, and the match tolerance was set to 0.01 (**Supplementary Figure 1**) (20). Two-independent samples t-test and the χ^2^ test were used to analyze the clinicopathologic characteristics between the LRH and ARH. DFS and OS after surgery were estimated using the Kaplan–Meier method, and a *p*-value < 0.05 was considered statistically significant. The log-rank test was used to compare the risk of developing recurrence and the risk of death between the two groups over the time (21). Cox proportional risk regression models were used to estimate the hazard ratios (HRs) and 95% confidence intervals (CIs) for the effect of surgical approaches on the OS and DFS (22). Statistical analysis was performed using GraphPad Prism version 6.0 (GraphPad Software, San Diego, CA, USA) and IBM-Microsoft SPSS version 22.0 (IBM Corporation, Armonk, NY, USA).

## Results

### Patient Characteristics Before and After Propensity-Matching

**Figure 1:**
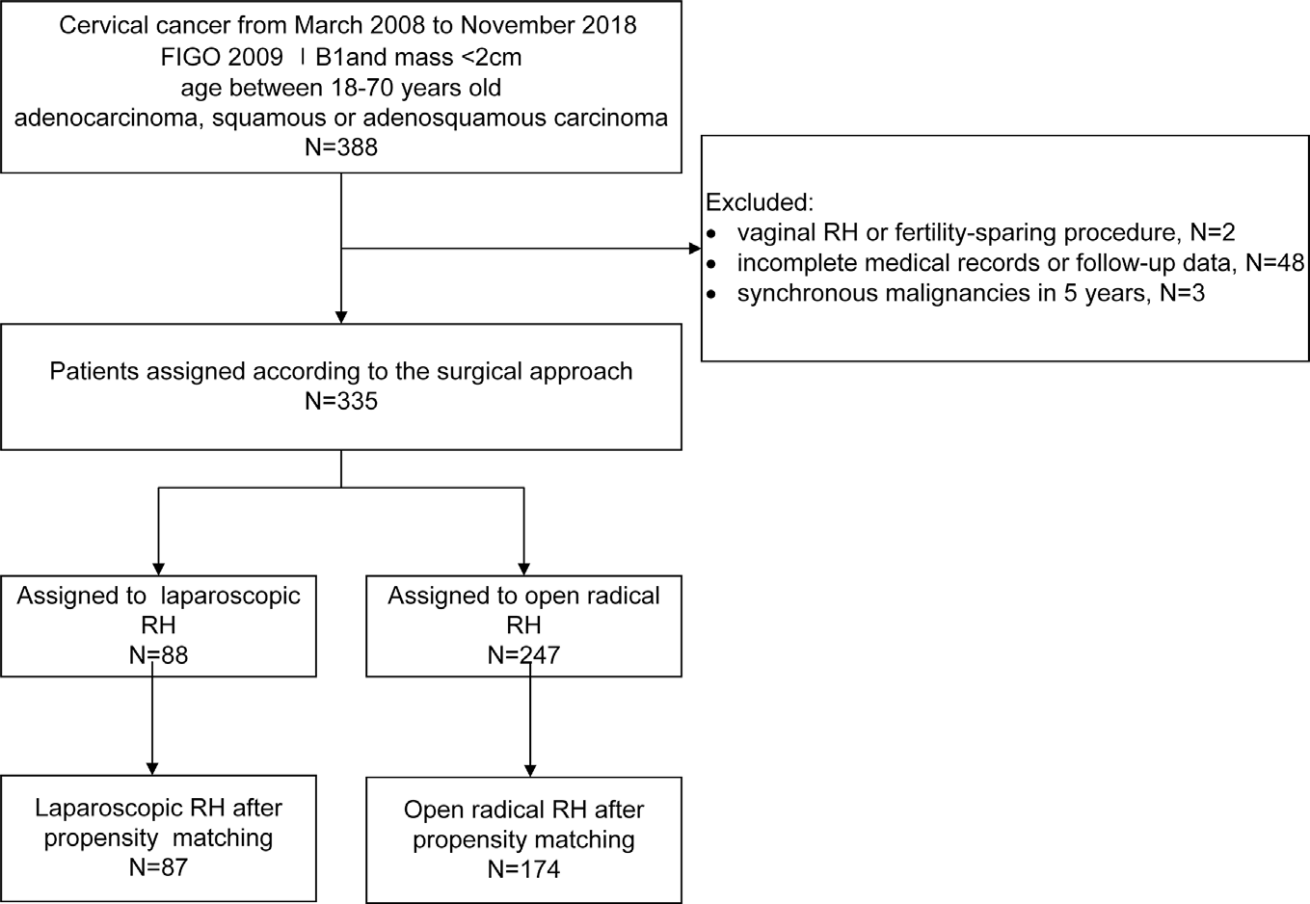
Flow diagram of recruitment and exclusions.

Over the study period, 335 patients met our inclusion and exclusion criteria. Among them, 247 patients underwent laparotomy and 88 underwent laparoscopy surgery (**Figure 1**). Patients in the laparoscopy group were propensity-matched 1:2 with those in the open RH group. After propensity score matching, 261 patients (87 in the laparoscopic group and 174 in the open procedure) were included in the following analysis, and no significant differences between two groups were observed in baseline characteristics. The clinicopathologic characteristics of the two groups before and after propensity-matching are presented in **Table 1**. Those patients who underwent ARH were more likely to have deeper depth of cervical stromal invasion (*p* = 0.047) and poorer differentiation (*p* = 0.002).

**Table 1:** Clinicopathologic characteristics before and after propensity score matching.

Variables	Before propensity score matching			After propensity score matching		
	ARH	LRH	*p* value	ARH	LRH	*p* value
	247	88		174	87	
Age (year, Mean ± SD)	51.53 ± 10.31	49.35 ± 8.81	0.079	48.00 ± 14.00	49.00 ± 12.00	0.979
Histology (%)			0.153			0.388
Squamous	205 (82.99)	66 (75.00)		140 (80.46)	66 (75.86)	
Adenocarcinoma	32 (12.96)	19 (21.59)		27 (15.52)	18 (20.69)	
Adenosquamous	10 (4.05)	3 (3.41)		7 (4.02)	3 (3.45)	
Differentiation (%)			0.002*			0.676
G1/G2	92 (37.25)	34 (38.64)		58 (33.33)	30 (34.48)	
G3	110 (44.53)	24 (27.27)		77 (44.25)	34 (39.08)	
Unknown/missing	45 (18.20)	30 (34.10)		39 (22.40)	23 (26.44)	
Surgical margin (%)			1.000			1.000
Negative	245 (99.19)	87 (98.86)		173 (99.43)	86 (98.85)	
Positive	2 (0.81)	1 (1.14)		1 (0.57)	1 (1.15)	
Pelvic lymph nodes (%)			0.340			1.000
Negative	227 (91.90)	84 (95.45)		166 (95.40)	83 (95.40)	
Positive	20 (8.10)	4 (4.55)		8 (4.60)	4 (4.60)	
LVSI (%)			0.638			0.856
Negative	199 (80.57)	74 (83.15)		148 (85.06)	73 (83.91)	
Positive	48 (19.43)	15 (16.85)		26 (14.94)	14 (16.09)	
DCSI (%)			0.047*			0.974
Inner 1/3	128 (51.82)	59 (67.05)		118 (67.82)	58 (66.67)	
Medium 1/3	72 (29.15)	17 (19.32)		32 (18.39)	17 (19.54)	
Outer 1/3	47 (19.03)	12 (13.64)		24 (13.79)	12 (13.79)	
Parametrial involvement (%)			0.570			
No	244 (98.79)	88 (100.00)		174 (100.00)	87 (100.00)	
Yes	3 (1.21)	0 (0.00)				
Adjuvant treatment given (%)			0.370			0.605
No	150 (60.73)	55 (62.50)		117 (67.24)	54 (62.07)	
Radiotherapy	30 (12.15)	5 (5.68)		14 (8.05)	5 (5.75)	
Chemotherapy	35 (14.17)	15 (17.05)		23 (13.22)	15 (17.24)	
Concomitant chemoradiotherapy	32 (12.96)	13 (14.77)		20 (11.49)	13 (14.94)	
SCC before surgery (Mean ± SD)			0.406			0.846
	1.00 ± 0.70	0.90 ± 1.00		1.00 ± 0.50	0.90 ± 1.00	

ARH, abdominal radical hysterectomy; LRH, laparoscopic radical hysterectomy; LVSI, lymphovascular space invasion, DCSI, depth of cervical stromal invasion; G1, well differentiated; G2, moderately differentiated; G3, poorly differentiated; LVSI, lymph vascular space invasion. Values are presented as mean ± standard deviation or number (%). *p < 0.05, statistically significant.

### Recurrence and Survival in Propensity-Matched Cohort

**Figure 2:**
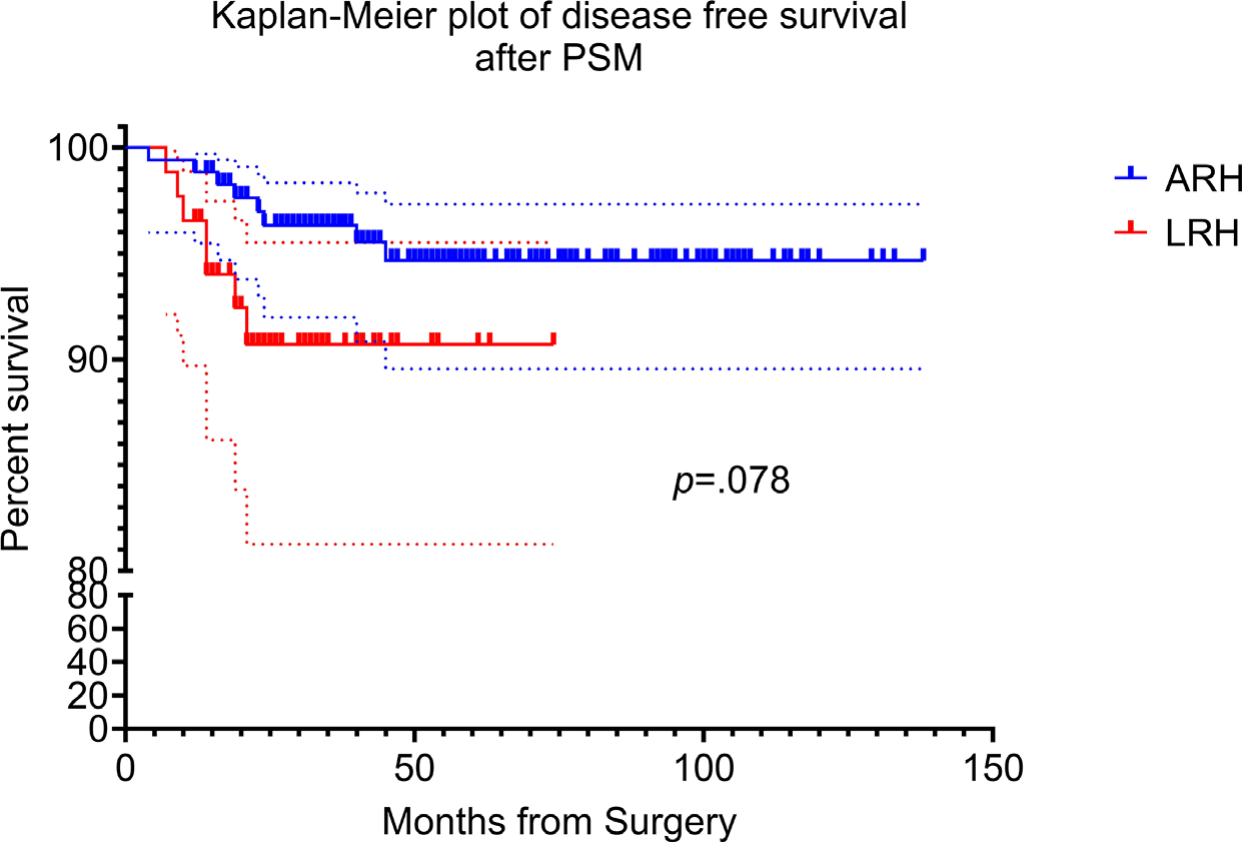
Kaplan–Meier disease free survival curves for laparoscopic radical hysterectomy and abdominal radical hysterectomy. The disease-free survival (DFS) rate of ARH and LRH after propensity score matching.

The median follow-up time was 42 months (range from 12 to 138 months). In the propensity matching cohort, there were eight patients underwent ARH experienced recurrence, which gives a recurrence rate of 4.6% (8/174). Disease relapsed in seven patients in laparoscopy group, for which the recurrence rate was 8.0% (7/87). Two-year and 5-year DFS was 97.1% versus 92.0% (*p* = 0.060) and 95.4% versus 92.0% (*p* = 0.260) for the open versus laparoscopic groups, respectively. Interestingly, Kaplan–Meier analysis indicated that patients in the LRH group showed tendency to suffer recurrence (HR, 2.838; 95% CI, 0.888–9.032; log-rank p = 0.078), even though there was no statistics difference between the two groups. Kaplan–Meier plot of DFS after PSM are presented in **Figure 2**.

**Figure 3:**
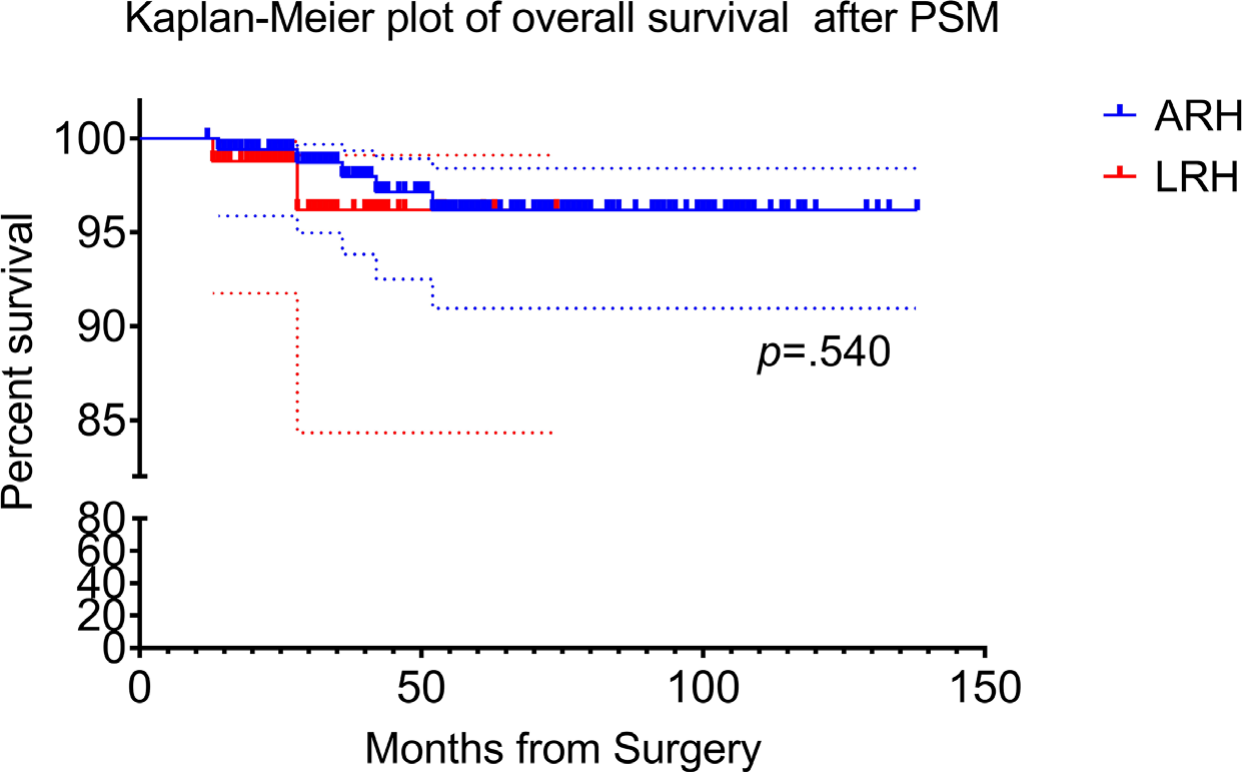
Kaplan–Meier survival curves for laparoscopic radical hysterectomy and abdominal radical hysterectomy. The overall survival (OS) rate of ARH and LRH after propensity score matching.

There were five patients or 2.9% (5/174) died in open surgery group and the corresponding percentage in laparoscopy group was 2.3% (2/87). Two-year and 5-year OS was 99.4% versus 97.7% (*p* = 0.218) and 97.1% versus 97.7% (*p* = 0.787) for the open versus laparoscopic groups, respectively. Kaplan–Meier analysis showed no difference in OS between the two groups in propensity score weighting cohort (HR, 1.823; 95% CI, 0.267–12.44; log-rank *p* = 0.540). Kaplan–Meier plot of OS after PSM are presented in **Figure 3**.

### Univariable and Multivariable Regression Analysis for Prognostic Factors

We performed univariate and multivariate Cox analysis to investigate the comprehensive prognostic factors for RFS (**Table 2**) and OS (**Table 3**). In univariable regression analysis of the matched cohort histology subtype adenosquamous (HR, 9.619; 95% CI, 2.545–36.353; *p* = 0.001) and positive pelvic lymph node (HR, 5.593; 95% CI, 1.577–19.835; *p* = 0.008) were potentially predictive factors of prognosis for RFS. The multivariate analysis model revealed histology subtype adenosquamous (HR, 8.919; 95% CI, 1.978–40.227; *p* = 0.004), positive pelvic lymph node (HR, 5.593; 95% CI, 1.577–19.835; *p* = .008) as well as laparoscopic operation procedure (HR, 3.789; 95% CI, 1.143–12.559; *p* = 0.029) were potentially predictive factors of DFS. Univariate Cox proportional hazard regression analysis revealed that positive pelvic lymph node (HR, 8.439; 95% CI, 1.637–43.504; *p* = 0.011), histology subtype adenosquamous (HR, 13.132; 95% CI, 1.187–145.267; *p* = 0.036) and adenocarcinoma (HR, 11.074; 95% CI, 2.019–60.733; *p* = 0.006) were predictors of OS. Furthermore, the multivariate survival analysis model revealed that the adenosquamous (HR, 17.662; 95% CI, 1.837–169.853; *p* = 0.013), adenocarcinoma (HR, 20.647; 95% CI, 1.234–345.376; *p* = 0.035) and positive pelvic lymph node (HR, 11.372; 95% CI, 1.890–68.440; *p* = 0.008) were potentially predictive factors of OS.

**Table 2:** Factors Associated with Recurrence-Free Survival.

Characteristics	Univariate		Multivariate	
	HR (95% CI)	*P*	HR (95% CI)	*P*
Histology				
Squamous	Reference		Reference	
Adenocarcinoma	2.457 (0.739–8.171)	0.143	2.536 (.618–10.404)	0.196
Adenosquamous	9.619 (2.545–36.353)	0.001*	8.919 (1.978–40.227)	0.004^*^
Surgery Approach				
Open	Reference		Reference	
Laparoscope	1.405 (0.143–3.145)	0.088	3.789 (1.143–12.559)	0.029*
Parametrial Involvement				
No	Reference			
Yes	5.169 (0.699–38.231)	0.108		
Pelvic lymph node				
Negative	Reference		Reference	
Positive	5.593(1.577–19.835)	0.008*	4.716 (1.067–20.8430)	0.041*
Surgical Margin				
Negative	Reference			
Positive	0.049 (0.000–400413)	0.814		
LVSI				
Negative	Reference			
Positive	0.739 (0.208–2.619)	0.639		
DCSI				
Inner 1/3	Reference			
Medium 1/3	0.569 (.151–2.147)	0.406		
Outer 1/3	1.056 (.236–4.718)	0.944		
Differentiation				
G1/G2	Reference			
G3	1.168 (0.515–2.649)	0.709		
Unknown/missing	0.525 (0.146–1.881)	0.322		
Adjuvant Therapy				
No	Reference			
Radiotherapy	0.819 (0.177–3.793)	0.799		
Chemotherapy	2.795 (0.995–7.857)	0.984		
Chemoradiotherapy	1.612 (0.295–8.806)	0.581		

LVSI, lymphovascular space invasion, DCSI, depth of cervical stromal invasion; G1, well differentiated; G2, moderately differentiated; G3, poorly differentiated.

*P < 0.05.

**Table 3:** Factors Associated with Overall Survival.

Characteristics	Univariate		Multivariate	
	HR (95% CI)	*P*	HR (95% CI)	*P*
Histology				
Squamous	Reference		Reference	
Adenocarcinoma	11.074 (2.019–60.733)	0.006*	20.647 (1.234–345.376)	0.013*
Adenosquamous	13.132 (1.187–145.267)	0.036*	17.662 (1.837–169.853)	0.035*
Surgery Approach				
Open	Reference			
Laparoscope	1.694 (0.309–9.296)	0.544		
Parametrial Involvement				
No	Reference			
Yes	0.848 (0.000–43)	0.767		
Pelvic lymph node				
Negative	Reference			
Positive	8.439 (1.637–43.504)	0.011*	11.372 (1.890–68.440)	0.008*
Surgical Margin				
Negative	Reference			
Positive	.049 (0.000–5.48E15)	0.880		
LVSI				
Negative	Reference			
Positive	1.083 (0.130–9.001)	0.941		
DCSI				
Inner 1/3	Reference			
Medium 1/3	2.325 (0.388–13.916)	0.355		
Outer 1/3	2.979 (0.497–17.838)	0.232		
Differentiation				
G1/G2	Reference			
G3	0.932 (0.178–2.247)	0.478		
Unknown/missing	0.343 (0.041–2.850)	0.322		
Adjuvant Therapy				
No	Reference			
Radiotherapy	0.529 (0.055–5.089)	0.582		
Chemotherapy	0.000 (.000)	0.986		
Chemoradiotherapy	2.471 (.257–23.772)	0.433		
Relapse				
No	Reference			
Yes	1.000 (.024–42.036)	1.000		

LVSI, lymphovascular space invasion, DCSI, depth of cervical stromal invasion; G1, well differentiated; G2, moderately differentiated; G3, poorly differentiated. *P < 0.05.

### The Pattern of Recurrence

All the recurrence occurred within two years after operation in the laparoscopy group, while in the open surgery group, five cases relapsed within 2 years and the other three cases recured within 5 years. When it comes to the recurrence type, most of the cases in the laparoscopic group suffered pelvic recurrence (6/7, 85.7%), and one case suffered vaginal stump recurrence. In the laparotomy group, two cases experienced vaginal stump recurrence, four cases experienced hematogenous recurrences (one case liver and lung recurrences, one case liver and two cases lung), and two cases suffered pelvic recurrence.

## Discussion

Although the safety of minimal invasive surgery in cervical cancer was questioned since the published result of LACC trial in 2018 (23), its advantages are undeniable. These advantages include less intraoperative blood loss, a shorter length of hospital stay, faster bowel and bladder function recovery, and a lower risk of postoperative complications (24, 25). Gynecological oncologists are trying to select patients with specific characteristics, who might benefit from minimal invasive surgery (26). Tumor dimension is one of the most studied specific characteristics. A consensus has been reached that there was no distinct advantage of LRH over ARH in tumors diameter >2 cm (11). However, the exact effect of surgical approach on oncological outcomes in patients with tumor diameter <2 cm is still controversial, and the related studies are limited.

Some studies found similar hazards of recurrence and death in both subgroups. Kim et al. reported that minimal invasive surgery did not influence PFS of stage IB1 patients with cervical mass ≤2 cm on pre-operative MRI (14). No difference in DFS was noted between robotic and open RH in cervical cancer tumor size ≤2 cm in a Sweden cohort (17). These results were supported by a multicenter retrospective study published by Chinese researchers (15). Recently, several studies reached the conclusion that minimally invasive RH had inferior DFS even for tumors that have size less than 2 cm. A multi-institutional retrospective study performed in the United States found that patients with tumor size ≤2 cm on final pathology had a higher recurrence rate in the minimally invasive approach (27). A Korean Gynecologic Oncology Group Study reached conclusion that LRH was associated with inferior DFS among patients with IB–IIA and tumor size <2 cm (16). There has been no widely accepted conclusion on the exact effect of surgical approach on oncological outcomes in tumor diameter <2 cm so far. On the other hand, there is a lack of uniformity in definition between different studies (i.e., tumor size based on MRI, clinical examination, or pathology; lesion diameter <2 cm or ≤2 cm; Da Vinci Robotic Surgery included or excluded; 3D laparoscopy or not; FIGO 2009 or 2018). In this study, we analyzed the clinical data from our center to explore the safety of LRH in FIGO 2009 stage IB1 cervical cancer patients with tumor diameter <2 cm in preoperative imaging exam. We concluded that LRH was associated with higher risk of recurrence than ARH and there is no difference between two groups in OS.

The multivariate analysis revealed that histology subtype adenosquamous, positive pelvic lymph node as well as laparoscopic operation procedure were potentially predictive factors of DFS. Adenosquamous, adenocarcinoma, and positive pelvic lymph node were potentially predictive factors for OS. We included those variables that were reported as risk factors for recurrence in the multivariate analyses (28, 29). Several variables showed no association with survival in univariate analysis in our study, including LVSI, depth of invasion, tumor grade, surgical margins, etc., which are typically associated worse outcomes. This situation might be explained by the small sample size and/or the uneven distribution between subgroups. Meanwhile, it is undeniable that these factors might, to a certain extent, influence the route of surgery. Doctors are more likely to perform open surgery on those patients with poor differentiation, deeper invasion and LVSI. There might be inter-operator variation in surgical treatment of cervical cancer between different surgeons.

The diameter of the tumor was measured *via* preoperative pelvic imaging evaluation according to MRI or CT in our study. Preoperative imaging assessment was more valuable and practical in clinical practice, and it is an important factor for surgeons to decide the route of surgery. There are still some differences between the preoperative imaging (CT or MRI) and the pathologic report. Although postoperative pathology could be interfered by preoperative conization and specimen treatment, it is still the gold standard of final diagnosis and stage. We had encountered the patients who had been underestimated by preoperative imaging assessment. How to accurately predict the tumor size and stage before surgery is a valuable research field.

Our results indicated that cervical cancer patients with a lesion less than 2 cm, who underwent LRH, were more likely to experience recurrence than those underwent ARH. In our study, all the recurrence in the laparoscopic group occurred within 2 years after surgery, and most of the recurrence occurred in pelvic. Our result was similar with the results from a study in South Korea (14). There are several potential reasons contributing to the higher recurrence for LRH. The uterine manipulator and the exposure of cervical cancer to circulating CO2 might increase tumor spillage (23). Besides, the prolonged Trendelenburg position (30) might also influence the relapse of cancer. A constructive manner to limit the use of invasive uterine manipulator and the time interval of opening the vagina should be taken into consideration. Intraperitoneal exposure during minimally invasive surgery had a significantly worse prognosis than no intraperitoneal exposure. Intraperitoneal tumor exposure was an independent prognostic factor for worse survival (31). A novel fluorescence imaging‐based tool for feasible and direct visualization of peritoneal contamination during colpotomy might serve as a quality assessment tool for surgeons and surgical techniques (32). Specific measures were adopted by some surgeons to prevent tumor spillage during LRH, such as creation of a vaginal cuff, minimized handling of the uterine cervix, and bagging the specimen (33). Recently, a multicenter retrospective observational cohort study concluded that conization before radical hysterectomy was associated with improved DFS in FIGO 2009 stage IB1 cervical cancer, and no conization before radical hysterectomy was an independent factor for higher risk of recurrence (34). However, whether conization before surgery would influence the oncologic outcomes between laparoscopic and open radical hysterectomy is still unknown and is an interesting direction for further study.

The current study had several limitations. First, this is a retrospective study. The heterogeneity differences between treatment groups still existed, even though propensity score matching was performed. Second, there might be inter-operator variation in surgical treatment of cervical cancer between different surgeons. Third, the sample size is small and the distribution of subgroup is uneven. Prospective multicenter studies are still needed to confirm our findings. Fourth, there might be some difference between the preoperative imaging modality (CT or MRI) and the actual pathologic tumor size. Pathological tumor size should be taken into consideration in future study.

In conclusion, we observed that cervical cancer patients with a lesion less than 2 cm might be more likely to have recurrence in LRH group than those taken ARH. Further randomized controlled perspective trials are needed to explore the advantages and disadvantages of the adoption of minimally invasive techniques in the treatment of cervical cancer patients with a lesion less than 2 cm.

## Data Availability Statement

The raw data supporting the conclusions of this article will be made available by the authors, without undue reservation.

## Author Contributions

XC performed data analysis, reviewed the literature and drafted the article. JY collected clinical data. HYZ and YH designed the study and finalized the paper. HQZ and YH provided suggestions to improve it. All authors contributed to the article and approved the submitted version. 

## Conflict of Interest

The authors declare that the research was conducted in the absence of any commercial or financial relationships that could be construed as a potential conflict of interest.

## Supplementary Material

Supplementary Figure 1 Diagram of propensity score matching.
