# Supplementary figures and images for: Laparoscopic Radical Hysterectomy Results in Higher Recurrence Rate Versus Open Abdominal Surgery for Stage IB1 Cervical Cancer Patients With Tumor Size Less Than 2 Centimeter: A Retrospective Propensity Score-Matched Study
Source: Front Oncol. 2021 Jun 10;11:683231. doi: 10.3389/fonc.2021.683231 (PMC8222693; doi:10.3389/fonc.2021.683231)

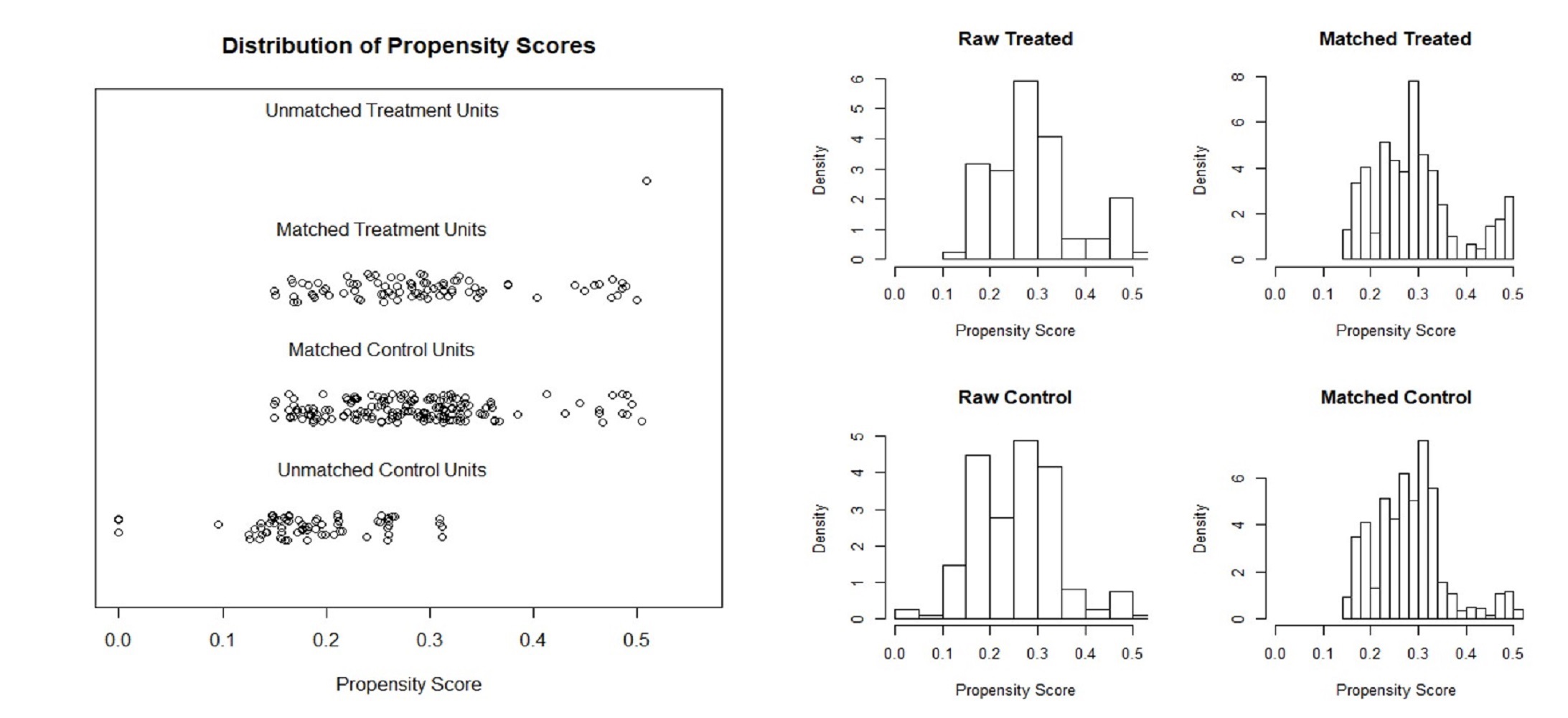

Supplement: Supplementary Figure 1 — Diagram of propensity score matching. [file Image_1.jpeg]
